# A mechanism for sequence specificity in plant‐mediated interactions between herbivores

**DOI:** 10.1111/nph.14328

**Published:** 2016-11-29

**Authors:** Wei Huang, Christelle A. M. Robert, Maxime R. Hervé, Lingfei Hu, Zoe Bont, Matthias Erb

**Affiliations:** ^1^ Institute of Plant Sciences University of Bern Altenbergrain 21 Bern 3013 Switzerland; ^2^ INRA UMR1349 IGEPP Le Rheu F‐35653 France

**Keywords:** aboveground−belowground interactions, *Diabrotica virgifera virgifera*, induced resistance, physiological canalization, plant–herbivore interactions, *Spodoptera frugiperda*, volatile organic compounds, *Zea mays*

## Abstract

- Herbivore communities are shaped by indirect plant‐mediated interactions whose outcomes are strongly dependent on the sequence of herbivore arrival. However, the mechanisms underlying sequence specificity are poorly understood.
- We examined the mechanisms that govern sequence‐specific effects of the interaction between two specialist maize herbivores, the leaf feeder *Spodoptera frugiperda* and the root feeder *Diabrotica virgifera virgifera*. In the field, *S. frugiperda* reduces *D. v. virgifera* abundance, but only when it arrives on the plant first.
- In behavioral experiments, *D. v. virgifera* larvae continued feeding on plants that they had infested before leaf infestation, but refused to initiate feeding on plants that were infested by *S. frugiperda* before their arrival. Changes in root‐emitted volatiles were sufficient to elicit this sequence‐specific behavior. Root volatile and headspace mixing experiments showed that early‐arriving *D. v. virgifera* larvae suppressed *S. frugiperda‐*induced volatile repellents, which led to the maintenance of host attractiveness to *D. v. virgifera*.
- Our study provides a physiological and behavioral mechanism for sequence specificity in plant‐mediated interactions and suggests that physiological canalization of behaviorally active metabolites can drive sequence specificity and result in strongly diverging herbivore distribution patterns.

## Introduction

Interspecific competition influences the structure, function and stability of natural and agricultural ecosystems (Loreau & de Mazancourt, 2013). For herbivorous insects, interspecific competition can occur through direct interference or through plant‐mediated, indirect effects (Denno *et al*., 1995). A growing number of studies show that plant‐mediated, indirect effects are the most common form of interspecific competition between herbivores (Ohgushi, 2005; Kaplan & Denno, 2007; Xiao *et al*., 2012; Huang *et al*., 2013) and that they act as driving forces of herbivore community composition in nature (Kaplan & Denno, 2007; Poelman & Dicke, 2014; Stam *et al*., 2014).

The outcome of plant‐mediated interactions between herbivores is determined by a number of factors, including the identity of the attacking herbivore, the identity of the plant and the identity of the responding herbivore (Johnson & Agrawal, 2005; Wurst & van der Putten, 2007; Xiao *et al*., 2012; Huang *et al*., 2014). Recently, the sequence of arrival was also identified as an important factor: depending on which species arrives first, the effect of one herbivore on the other can change drastically. Soler *et al*. (2012), for instance, observed that *Pieris brassicae* caterpillars grew bigger when feeding on *Brassica oleracea* plants if the plants were infested by *Brevicoryne brassicae* aphids before the arrival of *P. brassicae*, but not if both herbivores attacked the plant simultaneously. A recent meta‐analysis of interactions between leaf‐ and root‐feeding herbivores identified the sequence of arrival as a strong predictor for the directionality of effects for this type of plant‐mediated interaction (Johnson *et al*., 2012).

To date, several physiological hypotheses have been proposed that may explain sequence specificity (Erb *et al*., 2011a; Stam *et al*., 2014): plant‐mediated feedback loops, overriding induction effects and physiological canalization. Plant‐mediated feedback loops occur if two herbivores sharing a host plant influence each other reciprocally (Soler *et al*., 2012): a first arriving herbivore could then influence the behavior and damage patterns of a second arriver by inducing physiological changes in the plant, which, as a consequence, would change the plant‐mediated impact of the second herbivore on the first herbivore and thereby lead to sequence‐specific patterns. Overriding effects occur if one herbivore elicits a plant response that is much stronger than the response elicited by the other herbivore and thereby determines the resulting interaction (Stam *et al*., 2014). Physiological canalization is a phenomenon where plant responses are determined by the first arriving herbivore (Viswanathan *et al*., 2007). By suppressing the response that is normally elicited by a second herbivore, physiological canalization can lead to sequence‐specific effects.

Behavioral mechanisms may also lead to sequence specificity (Erb *et al*., 2011a; Karban, 2011). Asymmetrical host acceptance, for instance, refers to situations where a herbivore is less likely to start feeding on a new host plant than to continue feeding on a colonized host. This is a common pattern for sedentary herbivores such as miners and gall feeders and may lead to sequence‐specific effects by modulating the behavior of a herbivore differently, depending on whether it is arriving on a host plant second or whether it is already established when another herbivore arrives.

Plant physiological and herbivore behavioral mechanisms are not mutually exclusive. Asymmetrical host acceptance, for instance, may be favored by plant‐mediated feedback loops, overriding effects or physiological canalization. For example, a first arriving herbivore may negatively impact a second herbivore, which may decrease the capability of the second herbivore to induce volatile repellents, and in turn render the plant more attractive to the first herbivore. Furthermore, a first herbivore may trigger strong physiological changes in the plant which may render it attractive to itself irrespective of the potentially unattractive changes that are induced by a second arriving herbivore. Finally, a first herbivore may change the plant's physiology in a way that makes it unresponsive to the second herbivore, which may lead to the suppression of an otherwise unattractive physiological change. To date, the contributions of the different physiological and behavioral mechanisms and their combinations to sequence specificity have not been tested experimentally. As a consequence, the drivers of sequence specificity in indirect, plant‐mediated interactions are not well understood.

Here, we analyzed potential mechanisms leading to sequence specificity by studying the effect of attack by the leaf‐feeding larvae of *Spodoptera frugiperda* on the root‐feeding larvae of *Diabrotica virgifera virgifera* sharing maize (*Zea mays*) as a common host plant. Both herbivores occur on cultivated maize and its wild ancestors and cause severe damage in both agricultural and natural systems (Branson & Krysan, 1981; O'Day, 1998). They overlap spatially and temporally in the field, with their sequence of arrival varying considerably with climatic conditions and locations (Branson, 1976; O'Day, 1998). Our previous study within the same system revealed that *S. frugiperda* larvae significantly reduce the number of *D. v. virgifera* larvae feeding on maize roots in the field, but only when *S. frugiperda* larvae arrive first (Erb *et al*., 2011a). Subsequent experiments showed that maize root systems of plants which are attacked by leaf‐feeding caterpillars become highly unattractive to *D. v. virgifera* larvae, and that this effect is mediated by long‐ and short‐distance host acceptance cues (Robert *et al*., 2012a; Erb *et al*., 2015; Lu *et al*., 2016). By contrast, *D. v. virgifera* attack renders the plant highly attractive to conspecifics (Robert *et al*., 2012a) and reprograms the root metabolism to become more suitable for its own development (Robert *et al*., 2012b). Although *D. v. virgifera* reduces the performance of leaf‐feeders on maize under water‐limiting conditions, which may lead to plant‐mediated feedback loops (Erb *et al*., 2009, 2011b), we found no correlation between the amount of *S. frugiperda* leaf damage and the reduction of *D. v. virgifera* performance in our previous work (Erb *et al*., 2011a).

Based on these findings, we hypothesized that asymmetrical host acceptance may contribute to the sequence‐specific interaction patterns between *D. v. virgifera* and *S. frugiperda*, and that this asymmetrical acceptance behavior may be the result of either overriding effects or physiological canalization. We therefore conducted a series of behavioral experiments to explore the impact of the sequence of arrival on host plant attractiveness and acceptability for *D. v. virgifera* larvae. We then used a modified two‐by‐two‐arm olfactometer to test the influence of plant volatiles on the sequence‐specific behavior of *D. v. virgifera* and to distinguish between overriding effects and physiological canalization. Finally, we analyzed the changes in root volatiles elicited by the different arrival sequences to test for patterns of physiological canalization.

## Materials and Methods

### Plants and insects

Maize (*Zea mays* L.) seeds (hybrid Delprim) were obtained from Delley Seeds and Plants Ltd (Delley, Switzerland). They were sown individually in plastic pots (11 cm depth and 4 cm diameter) and placed in a glasshouse (26 ± 2°C; 14 : 10 h, light : dark; 55% relative humidity). Twelve days later (henceforth called day 0), plants with three fully developed leaves were used for experiments. Eggs of *Diabrotica virgifera virgifera* (LeConte) were obtained from the Agricultural Research Service, United States Department of Agriculture (Brookings, SD, USA) and larvae were reared on freshly germinated maize plants until use. *Spodoptera frugiperda* (J.E. Smith) eggs were obtained from the University of Neuchatel (Neuchâtel, Switzerland), and the hatching larvae were reared on a soy‐wheat germ diet (Bio‐Serv, Flemington, NJ, USA) until use.

### Plant treatments

**Figure 1:**
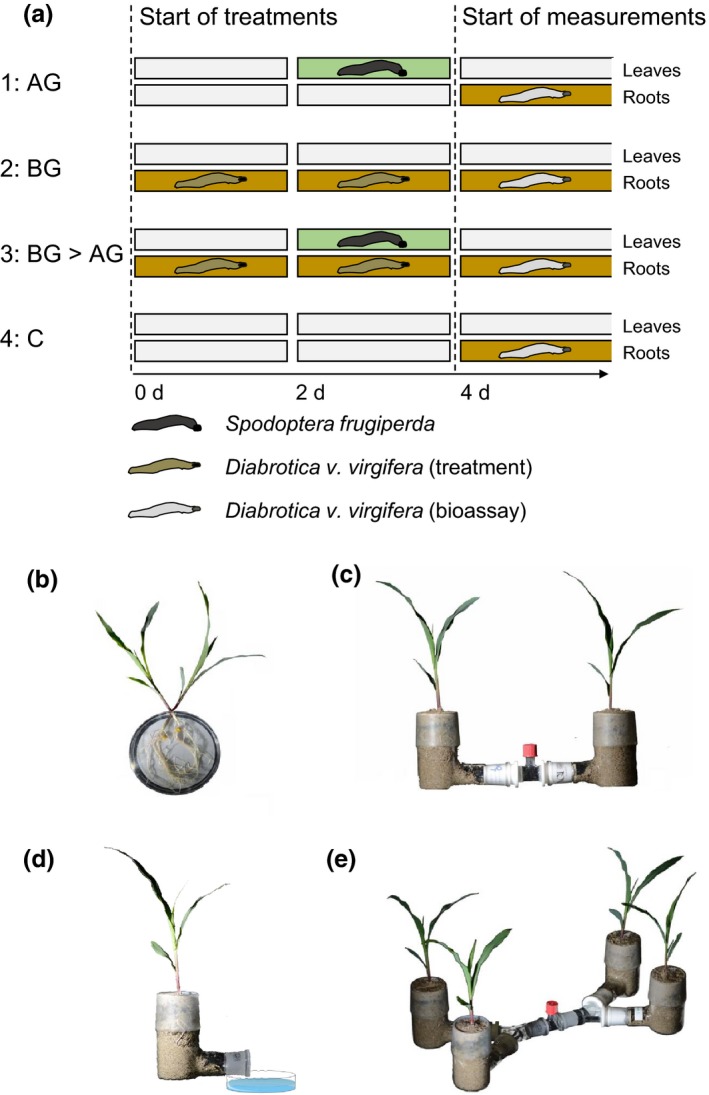
Overview of the experimental design and set‐ups used in this study. (a) Experimental treatments (infestation histories). To establish different sequences of arrival, second instar *Spodoptera frugiperda* larvae were added to the leaves, and second instar *Diabrotica virgifera virgifera* larvae were added to the roots of maize plants in different combinations. After 4 d of herbivore infestation, plants with different infestation histories were offered to *D. v. virgifera* larvae in choice and no‐choice experiments or were used for root volatile analyses. AG, aboveground *S. frugiperda* larval infestation; BG, belowground *D. v. virgifera* larval infestation; BG>AG, belowground infestation followed by aboveground infestation; C, control without herbivory. (b) Larval preference was measured by laying out the root systems of two plant on moist filter paper in large Petri dishes. (c) Volatile‐mediated larval preference was measured using a two‐arm belowground olfactometer. (d) Larval escape patterns were measured using a single L‐shaped glass pot and a water‐filled Petri dish to collect the escaping larvae. (e) Volatile mixing experiments were conducted using a two‐arm belowground olfactometer with two volatile sources attached to each arm of the central chamber. For more details about the different treatments and set‐ups, refer to the Materials and Methods section.

To establish different feeding sequences and herbivore combinations, plants were randomly assigned to one of four treatments (Fig. 1a): (1) aboveground herbivory (AG): 12 second‐instar *S. frugiperda* larvae were added to the leaves of each plant at day 2; (2) belowground herbivory (BG): six second‐instar *D. v. virgifera* larvae were added into a hole (9 cm depth and 0.5 cm diameter) in the soil at the base of each plant at day 0; (3) belowground attack followed by aboveground attack (BG > AG): six second‐instar larvae of *D. v. virgifera* were added to each plant at day 0, and 12 second‐instar larvae of *S. frugiperda* were added to each plant at day 2; (4) controls without herbivory (C). These treatments simulated a situation where *D. v. virgifera* larvae newly arrive on plants already infested with *S. frugiperda* (AG) or where they can continue feeding on maize plants that are infested by conspecifics alone (BG) or by conspecifics that arrived before the arrival of the leaf feeder (BG > AG) (Fig. 1a). As *D. v. virgifera* larvae refuse to feed on plants that have previously been attacked by *S. frugiperda* (Robert *et al*., 2012a; Erb *et al*., 2015; Lu *et al*., 2016), an AG > BG treatment was not included in the experimental set‐ups.

To prevent above‐ and belowground herbivores from escaping, the aboveground parts (leaves of maize plants) were caged with transparent 1.5‐l plastic bottles with their bottoms removed that were placed upside‐down on the pots. Belowground parts (pots) were covered with aluminum foil. All plants were caged in the same way regardless of herbivore treatment. Furthermore, small holes were made in the soil of each plant regardless of *D. v. virgifera* infestation. Four days after the beginning of the different treatments (day 4), the plastic bottles and *S. frugiperda* larvae were removed. Then, the responding *D. v. virgifera* larvae were introduced into the system (Fig. 1b–e). Timing and herbivore densities were chosen to match earlier studies and to mimic natural occurrence patterns in the field (Erb *et al*., 2011a; Robert *et al*., 2012a).

### Influence of sequence of arrival on host plant acceptance by *D. v. virgifera*

In a first set of experiments, we tested the hypothesis that *D. v. virgifera* larvae may reject roots of plants that are previously infested with *S. frugiperda*, but may continue to feed on plants on which they were able to establish a suitable feeding environment before the arrival of the leaf‐feeder. We conducted experiments using three different set‐ups (Fig. 1b–d).

First, we tested the behaviour of *D. v. virgifera* using a Petri dish set‐up which allowed for direct root contact (Robert *et al*., 2012c) (Fig. 1b). The root systems of plants from the different treatment groups were gently washed with tap water. Plants were then paired in the following combinations: (1) C vs AG; (2) C vs BG; (3) C vs BG > AG. Root systems of the different plant pairs were placed on a moistened filter paper in a Petri dish (13.5 cm diameter and 2 cm depth), which had a gap (0.8 cm width and 2 cm height) in the side. The stems were laid into the gap, with the leaves remaining outside the Petri dish. Six second‐instar larvae were then added to the moistened filter paper. The larvae could move and feed freely on the plants within the Petri dish. The Petri dish was covered with aluminum foil to decrease the impact of light on the roots and insects. The position of the larvae was recorded at 0.5, 1.5, 3 and 5 h. Larvae that remained on the filter paper and did not choose a plant were counted as no choice. Each treatment combination was repeated 24–36 times.

Second, we specifically tested the contribution of volatile cues to the observed behavioral patterns. For this purpose, the same treatment combinations as in the first experiment were offered to *D. v. virgifera* larvae in two‐arm olfactometers as described previously (Robert *et al*., 2012a) (Fig. 1c). Before the beginning of the treatments, plants were transplanted individually into L‐shaped glass pots (11 cm depth and 5 cm diameter) with a horizontal connector at a height of 0.5 cm and filled with moist sand. At day 4, the horizontal connector of each glass pot was attached with one Teflon connector (29/32 to 24/29 mm) which contained a fine metal screen (2300 mesh; Small Parts Inc., Miami Lakes, FL, USA). Then, the two Teflon connectors were linked using a glass tube (24/29 mm; length 8 cm) with a vertical access port in the middle. To keep the root systems in the dark and to avoid visual cues for the larvae, the entire olfactometer was covered with aluminum foil. Twenty minutes after connecting the different odor sources, six second‐instar *D. v. virgifera* larvae were released into the access port of the glass tube. The larvae could move freely in the glass tube, but could not reach the roots of the plants. After 10 min, the olfactometer was disassembled and the number of larvae in each Teflon connector was recorded. Larvae that stayed in the central glass tube after 10 min were recorded as no choice. For each treatment combination, 18 independent replicates were carried out.

In a third experiment, we tested whether *D. v. virgifera* larvae are more likely to leave the rhizosphere environment of infested plants, even in the absence of an alternative host. For this purpose, plants were potted and infested in L‐shaped glass pots as described for the second experiment (Fig. 1d). Then, six second‐instar larvae were released directly at the entrance of the horizontal access port of each glass pot. The access port of the horizontal connector was not sealed so the larvae could move into the soil and start feeding or try to escape from the plant through the access port. The L‐pot was placed in a Petri dish filled with tap water at a height of 0.5 cm to catch escaping *D. v. virgifera* larvae without flooding the glass pot. The number of escaped larvae in the trap was recorded over 20 min. For each treatment, 12 replicates were carried out.

### Plant‐mediated feedback loops

To evaluate whether belowground attack by *D. v. virgifera* changes the pattern of aboveground damage by *S. frugiperda* larvae under the current experimental conditions, the leaves of plants from the different infestation treatments were collected at day 4, and total leaf area and damaged leaf area were measured for each plant using digimizer software (MedCalc Software bvba, Mariakerke, Belgium). Eighteen replicates per treatment were carried out.

### Overriding effects

To investigate whether an overriding signal may be responsible for the observed asymmetrical host acceptance of *D. v. virgifera* in the first set of experiments, we developed a two‐by‐two arm belowground olfactometer that allowed us to combine the volatile headspaces from two odor sources per arm (Fig. 1e). For this purpose, the two‐arm olfactometer set‐up was modified as follows. Two Teflon connectors attached to glass pots were linked using a ‘Y’ glass tube (24/29 mm; length 8 cm) at an angle of 60°. Then, two ‘Y’ glass tubes were connected to a central glass tube (24/29 mm; length 8 cm) with a vertical access port in the middle. This modification enabled us to attach two L‐shaped glass pots to each side of the release tubes and to test the preference of *D. v. virgifera* for two combinations of two mixed odor sources. The following treatment combinations were investigated using this set‐up: C + C vs C + AG; C+C vs C + BG, and C + C vs AG + BG. The olfactometer was disassembled and the number of larvae in each ‘Y’ glass tube was recorded after 10 min. We hypothesized that, if *D. v. virgifera* elicits an overriding signal, the AG + BG arms should be more attractive than the C + C arm. Eighteen replicates were performed for each treatment combination.

### Physiological canalization

To evaluate whether *D. v. virgifera* attack canalizes the root volatile response in a way that suppresses responsiveness to *S. frugiperda* infestation, we collected and analyzed root volatile profiles using solid‐phase micro‐extraction−gas chromatography−mass spectrometry (SPME‐GC‐MS). Plants were treated as described in the section ‘Plant treatments’ (Fig. 1a). Crown and primary roots were then washed with tap water and frozen in liquid nitrogen. Twelve plants per treatment were harvested, and the roots of two plants were pooled for analysis, resulting in six biological replicates. The crown and primary roots of each replicate were ground into a fine powder, and 50 mg of each root type was placed in a 10‐ml glass vial and sealed using Teflon tape (polytetrafluoroethylene). An SPME fiber (100‐μm polydimethylsiloxane coating; Supelco, Bellefonte, PA, USA) was then inserted into the vial for 60 min at 50°C. The incubated fibers were then immediately analyzed by GC‐MS (Agilent 7820A GC interfaced with an Agilent 5977E MSD, Palo Alto, CA, USA) following previously established protocols with a few modifications (Erb *et al*., 2011c). Briefly, the fiber was inserted into the injector port at 250°C and desorbed for 2 min. After fiber insertion, the column temperature was maintained at 60°C for 1 min and then increased to 250°C at 5°C min^−1^ followed by a final stage of 4 min at 250°C. The overall analysis time for each sample, including oven cooling, was 45 min. Furthermore, to eliminate the impact of background peaks, three glass vials without any plant material (blanks) were run using the same protocol. The resulting GC‐MS chromatograms were processed with progenesis qi (Nonlinear Dynamics, Newcastle, UK) using default settings for spectral alignment and peak picking. From the resulting matrix, all features that were presented in more than one blank were removed, resulting in 232 features. Features were assigned to individual compounds by retention time and peak shape matching and identified using the NIST search 2.2 Mass Spectral Library (Gaithersburg, MD, USA) as well as retention time and spectral comparison with pure compounds.

### Data analysis

To examine host acceptance of *D. v. virgifera* in a Petri dish experiment, the number of larvae found on different herbivory treatment groups was analyzed using a Wald test applied to a generalized linear mixed model (GLMM) with a Poisson distribution. We considered plant treatment as a fixed factor, time as a covariate and the replicate as a random factor. Each plant combination (C vs AG, C vs BG and C vs BG > AG) was analyzed separately. Then, to compare the preference of *D. v. virgifera* between the different treatment groups, the number of larvae on infested plants (AG, BG and BG > AG) was analyzed using a likelihood ratio test applied to a generalized linear model (GLM) with a Poisson distribution. The models included herbivory as a fixed factor and time as a covariate. The preference of *D. v. virgifera* larvae in the olfactometer experiments and the number of escaped larvae in the escape experiment were analyzed in the same manner. To examine whether belowground attack by *D. v. virgifera* larvae changes the pattern of aboveground damage by *S. frugiperda* larvae, the relative and absolute leaf damage of *S. frugiperda* larvae was analyzed using independent sample *t*‐tests (BG vs BG > AG). The absolute leaf damage was estimated from the sum of leaf damaged area for each plant and the relative leaf damage was calculated as the sum of leaf damaged area/the sum of total leaf area × 100 for each plant. To examine the overall differences in volatile profiles, the relative abundance of the detected features was subjected to redundancy analysis (RDA) using the different treatments as a unique explanatory variable. Monte Carlo tests with 999 permutations were then used to test for significant differences between treatments. For more detailed, compound‐specific analyses, the different features were assigned to individual compounds, and the relative abundances of the individual compounds, which corresponds to the sum of the signal intensities of the individual features, were analyzed by one‐way ANOVAs followed by least square mean post hoc tests for pairwise comparisons, including false discovery rate (FDR) corrections (Benjamini & Hochberg, 1995). All analyses were conducted using R 3.2.0 (R Foundation for Statistical Computing, Vienna, Austria) with ‘car’, ‘lme4’, ‘lsmeans’, ‘vegan’ and ‘RVAideMemoire’ packages (Fox & Weisberg, 2011; Bates *et al*., 2015; Hervé, 2016; Lenth, 2016; Oksanen *et al*., 2016).

## Results

### *Diabrotica virgifera virgifera* rejects *S. frugiperda‐*infested plants only when arriving second

**Figure 2:**
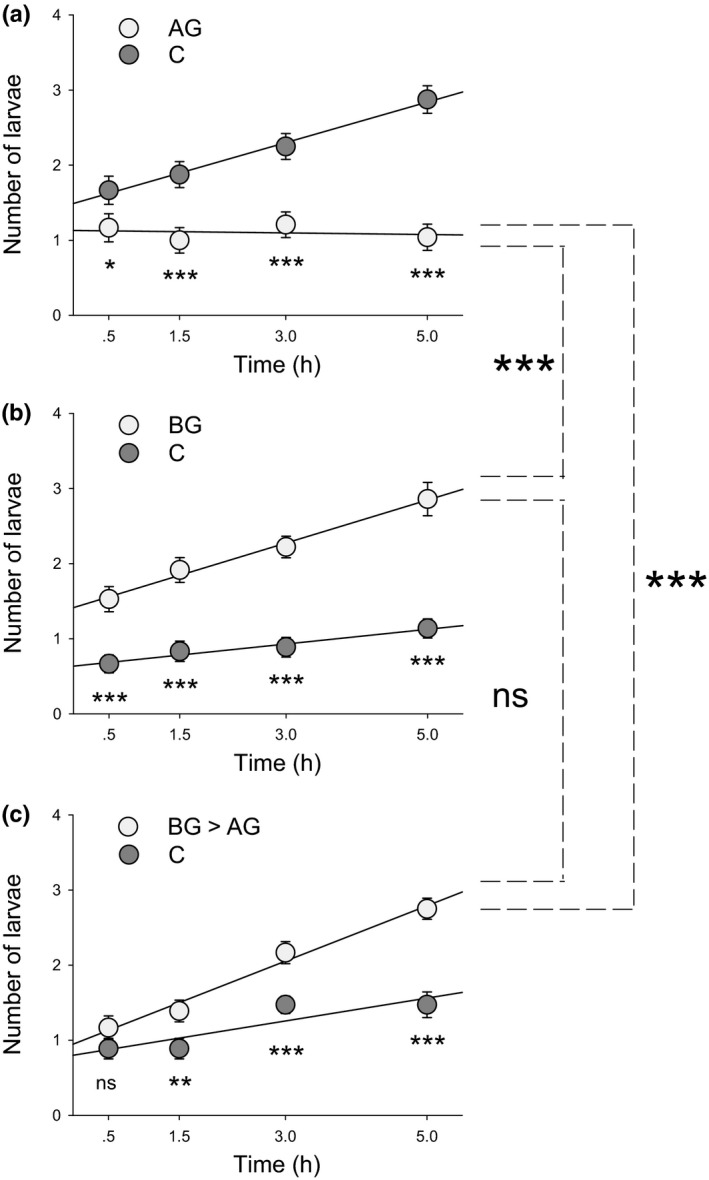
Sequence of arrival determines root attractiveness to *Diabrotica virgifera virgifera*. The number of *D. v. virgifera* larvae on the roots of plants with different infestation histories was measured in a Petri dish experiment. (a) *Diabrotica virgifera virgifera* choice between C and AG plants (*n *=* *24). (b) *Diabrotica virgifera virgifera* choice between C and BG plants (*n *=* *36). (c) *Diabrotica virgifera virgifera* choice between C and BG > AG plants (*n *=* *36). AG, aboveground *Spodoptera frugiperda* larval infestation; BG, belowground *D. v. virgifera* larval infestation; BG > AG, belowground infestation followed by aboveground infestation; C, control without herbivory. Values correspond to mean ± 1 SE. Asterisks indicate a significant difference in preference within each combination and time‐point (ns, nonsignificant; *, *P *<* *0.05; **, *P *<* *0.01; ***, *P *<* *0.001; GLMM). Differences in preference patterns between treatment combinations are depicted by dashed lines and asterisks on the right of the graph (ns, nonsignificant; ***, *P *<* *0.001; GLM).

In the Petri dish experiment, *D. v. virgifera* larvae strongly preferred the roots of control plants when offered uninfested vs leaf‐infested plants (χ^2^ = 30.753; *P *<* *0.001; Fig. 2a). By contrast, the larvae showed a strong preference for roots that had previously been infested with *D. v. virgifera* larvae over controls (χ^2^ **= 69.919; *P *<* *0.001; Fig. 2b). Roots that were infested with *D. v. virgifera* 2 d before the onset of *S. frugiperda* attack remained highly attractive (χ^2^ = 21.734; *P *<* *0.001; Fig. 2c). The number of responding *D. v. virgifera* larvae increased with experimental time (C vs AG: χ^2^ = 5.698; *P *=* *0.017; C vs BG: χ^2^ = 20.033; *P *<* *0.001; C vs BG > AG: χ^2^ = 35.964; *P *<* *0.001; Fig. 2). At the end of the experiment, 65%, 67% and 70% of *D. v. virgifera* larvae made a choice in C vs AG, C vs BG and C vs BG > AG, respectively. No significant interactive effects between time and treatment were found (C vs AG: χ^2^ = 3.515; *P *=* *0.061; C vs BG: χ^2^ = 0.135; *P *=* *0.713; C vs BG > AG: χ^2^ = 1.342; *P *=* *0.247). Overall, more *D. v. virgifera* larvae fed on BG and BG > AG roots than on AG roots (χ^2^ = 38.558; *P *<* *0.001; Fig. 2). No difference was found between the preference of *D. v. virgifera* for BG and BG > AG roots (*P *=* *0.064; Fig. 2).

**Figure 3:**
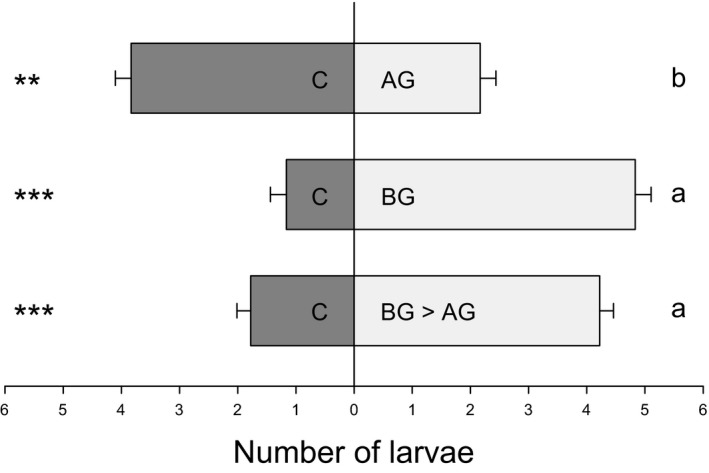
Volatile cues contribute to sequence‐specific preference patterns of *Diabrotica virgifera virgifera*. The number of *D. v. virgifera* larvae attracted to root volatiles of plants with different infestation histories was measured in a two‐arm olfactometer experiment. AG, aboveground *Spodoptera frugiperda* larval infestation; BG, belowground *D. v. virgifera* larval infestation; BG > AG, belowground infestation followed by aboveground infestation; C, control without herbivory. Values are mean ± 1 SE (*n *=* *18). Asterisks indicate a significant preference within each treatment combination (**, *P *<* *0.01; ***, *P *<* *0.001; GLMM). Different letters indicate significant differences between treatment combinations (*P *<* *0.05; GLM).

In the two‐arm olfactometer experiment, similar preference patterns were observed*. Diabrotica virgifera virgifera* larvae showed a strong preference for control plants over *S. frugiperda*‐infested plants (χ^2^ = 8.111; *P *<* *0.01; Fig. 3). By contrast, the larvae preferred plants that were previously infested with conspecifics over controls (χ^2^ = 34.177; *P *<* *0.001; Fig. 3). Plants infested with *D. v. virgifera* before *S. frugiperda* infestation remained highly attractive (χ^2^ = 16.849; *P *<* *0.001; Fig. 3). In this experiment, all larvae made a choice within 10 min. Overall, the larvae were more attracted to the roots that had been infested by conspecifics alone and conspecifics that had arrived before the arrival of the *S. frugiperda*, while they were less attracted to the roots that had been infested by *S. frugiperda* alone (χ^2^ = 20.396; *P *<* *0.001; Fig. 3). Again, BG and AG > BG treatments were not significantly different from each other (*P *=* *0.389; Fig. 3).

**Figure 4:**
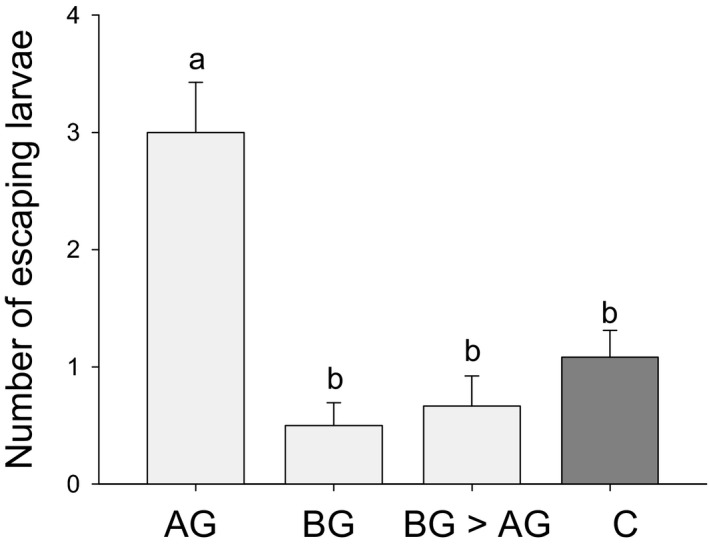
Stay‐or‐leave patterns of *Diabrotica virgifera virgifera* are determined by the sequence of arrival. The number of *D. v. virgifera* larvae leaving the rhizosphere of plants with different infestation histories was measured in an escaping experiment. AG, aboveground *Spodoptera frugiperda* larval infestation; BG, belowground *D. v. virgifera* larval infestation; BG > AG, belowground infestation followed by aboveground infestation; C, control without herbivory. Values are mean ± 1 SE (*n *=* *12). Different letters indicate significant differences between treatments (*P *<* *0.05; GLM).

When offered a single host plant, the number of escaping *D. v. virgifera* larvae differed significantly between treatments (χ^2^ = 32.112; *P *<* *0.001; Fig. 4). When offered a *S. frugiperda*‐infested plant, 50% of the larvae escaped from the rhizosphere within 20 min (Fig. 4). By contrast, < 18% of the larvae left the soil of control plants or plants that were previously infested with conspecifics (Fig. 4). A similar percentage of larvae chose to remain in the rhizosphere of plants that were infested with *D. v. virgifera* before *S. frugiperda* attack (Fig. 4).

### Plant‐mediated feedback loops are unlikely to explain *D. v. virgifera* behavior

There was no significant difference in relative (*t *=* *0.055; *P *=* *0.957) or absolute (*t *=* *1.236; *P *=* *0.225) damaged leaf area between plants that were infested with *D. v. virgifera* and plants with roots that were herbivore free (Supporting Information Fig. S1). These results suggest that the interaction between *D. v. virgifera* and *S. frugiperda* is highly asymmetrical and that plant‐mediated feedback loops are unlikely to play a major role in determining sequence‐specific responses of *D. v. virgifera*.

### *Diabrotica virgifera virgifera* does not produce an overriding attractive signal

**Figure 5:**
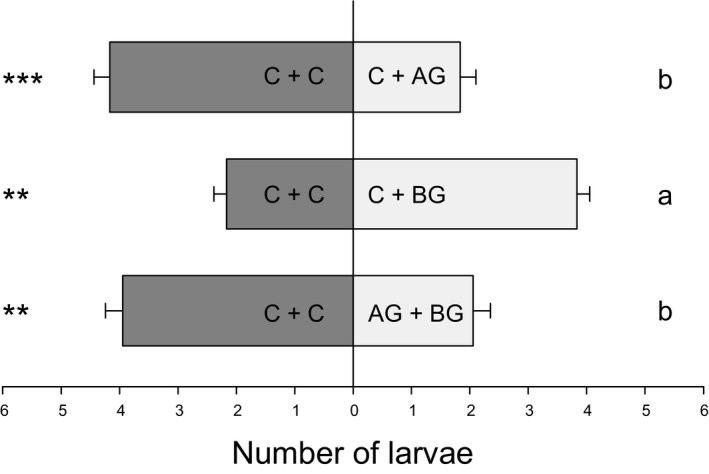
Acceptance of *Diabrotica virgifera virgifera* is determined by the additive changes in root volatiles. The number of *D. v. virgifera* larvae attracted by mixed root volatiles from plants with different infestation histories were measured in a volatile‐mixing experiment, with each arm containing two different volatile sources. AG, aboveground *Spodoptera frugiperda* larval infestation; BG, belowground *D. v. virgifera* larval infestation; C, control without herbivory. Values are mean ± 1 SE (*n *=* *18). Asterisks indicate a significant preference within choice combinations (**, *P *<* *0.01; ***, *P *<* *0.001; GLMM). Different letters indicate differences in preference patterns between treatments (*P *<* *0.05; GLM).

Similarly to the two‐arm olfactometer experiment, *D. v. virgifera* larvae significantly more often preferred to move to the side of the olfactometer containing two control plants rather than the arm leading to a control plant and an *S. frugiperda*‐infested plant (χ^*2*^ = 15.446; *P *<* *0.001; Fig. 5). The opposite was true for a combination of a control plant with a *D. v. virgifera*‐infested plant, which was attractive to the root feeder (χ^*2*^ = 8.111; *P *<* *0.01; Fig. 5). In contrast to the attractiveness of BG > AG plants observed in the two‐arm olfactometer experiment, however (Fig. 3), the mixed rhizosphere volatiles from an *S. frugiperda‐* and a *D. v. virgifera*‐infested plant were highly unattractive, and significantly more larvae moved to the control side (χ^*2*^ = 10.333; *P *<* *0.01; Fig. 5) than to the AG + BG side. All larvae made a choice within the first 10 min. Overall, the presence of plants that were infested by *S. frugiperda* significantly more often repelled *D. v. virgifera* (χ^2^ = 15.915; *P *<* *0.001; Fig. 5). This experiment falsifies the hypothesis that *D. v. virgifera* triggers an overriding attractant.

### *Diabrotica virgifera virgifera* feeding suppresses *S. frugiperda*‐induced root volatiles

**Figure 6:**
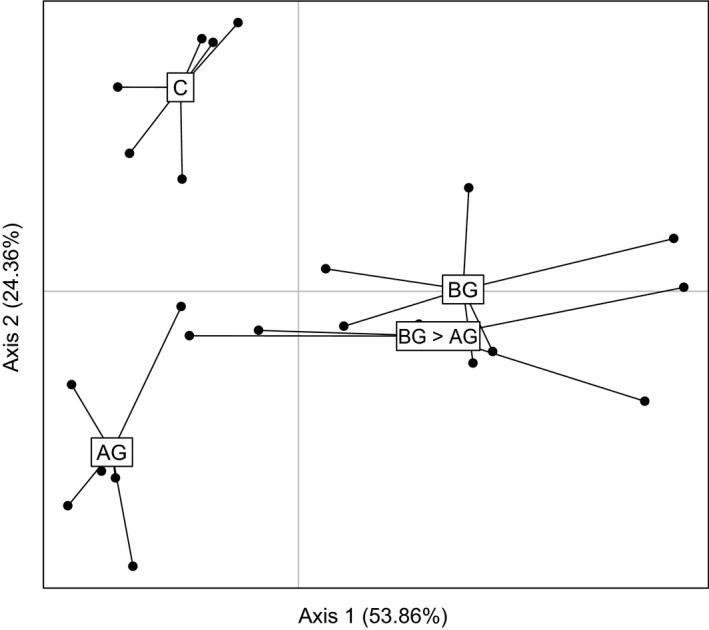
Infestation by *Diabrotica virgifera virgifera* canalizes the volatile response of maize roots. The results of a redundancy analysis (RDA) of the root volatile response to different sequences of *D. v. virgifera* and *Spodoptera frugiperda* feeding are shown. The first two axes explained 53.86% and 24.36% of the total variation, respectively. AG, aboveground *S. frugiperda* larval infestation; BG, belowground *D. v. virgifera* larval infestation; BG > AG, belowground infestation followed by aboveground infestation; C, control without herbivory. Data points represent individual replicates (*n *=* *6).

**Figure 7:**
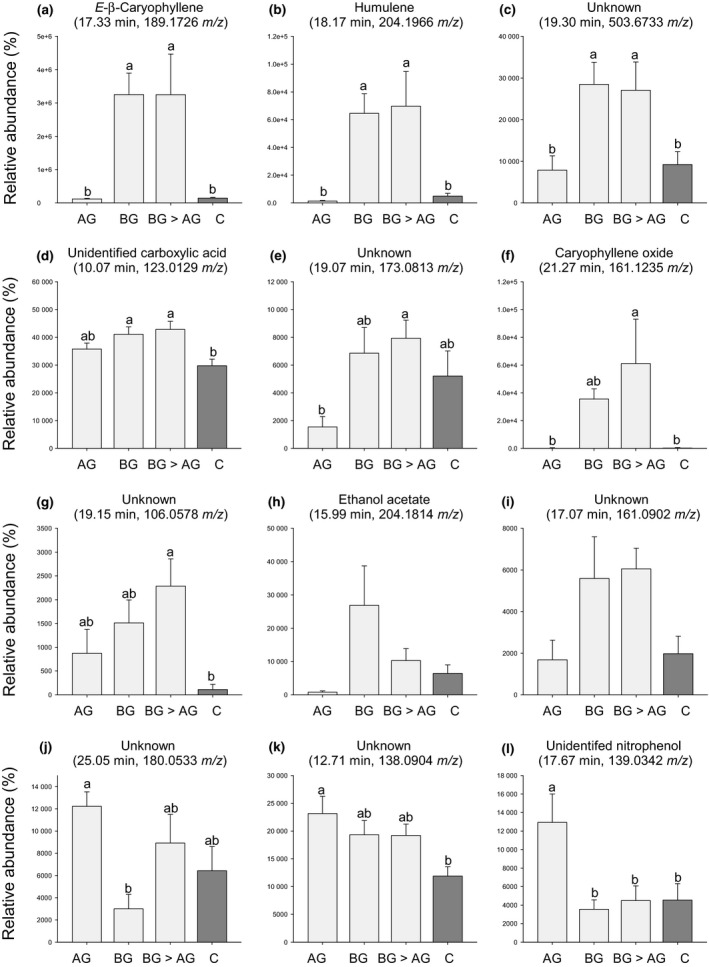
*Diabrotica virgifera virgifera* suppresses *Spodoptera frugiperda*‐induced root volatiles. The relative abundances of root volatiles in four treatments were measured using solid‐phase micro‐extraction (SPME) in combination with gas chromatograpy and mass spectrometry (GC‐MS). (a) *E‐*β‐Caryophyllene (17.33 min; 189.1726 *m/z*), (b) humulene (18.17 min; 204.1966 *m/z*), (c) unknown (19.30 min; 503.6733 *m/z*), (d) unidentified carboxylic acid (10.07 min; 123.0129 *m/z*), (e) unknown (19.07 min; 173.0813 *m/z*), (f) caryophyllene oxide (21.27 min; 161.1235 *m/z*), (g) unknown (19.15 min; 106.0578 *m/z*), (h) ethanol acetate (15.99 min; 204.1814 *m/z*), (i) unknown (17.07 min; 161.0902 *m/z*), (j) unknown (25.05 min; 180.0533 *m/z*), (k) unknown (12.71 min; 138.0904 *m/z*) and (l) unidentified nitrophenol (17.67 min; 139.0342 *m/z*). AG, aboveground *S. frugiperda* larval infestation; BG, belowground *D. v. virgifera* larval infestation; BG > AG, belowground infestation followed by aboveground infestation; C, control without herbivory. Values are mean ± 1 SE (*n *=* *6). Different letters indicate differences in relative abundance among treatments (*P *<* *0.05; LM).

In total, we detected 232 volatile features in the GC‐MS chromatograms. Redundancy analysis revealed that *S. frugiperda* and *D. v. virgifera* attack induced different volatile blends compared with control plants and compared with each other (AG vs C: *P *=* *0.008; BG vs C: *P *=* *0.008; BG>AG vs C: *P *=* *0.008; Fig. 6). The volatile profiles of plants that were induced by *D. v. virgifera* before *S. frugiperda* attack were indistinguishable from those of plants that were infested with *D. v. virgifera* alone (BG > AG vs BG: *P *=* *0.642; Fig. 6), but both were significantly different from those of plants that were infested with *S. frugiperda* alone (BG vs AG: *P *=* *0.008; BG > AG vs AG: *P *=* *0.008; Fig. 6). Analysis of variance revealed 12 volatile compounds whose abundance differed significantly between treatments (Fig. 7). Pairwise comparisons showed that four of these volatiles were significantly induced by *D. v. virgifera* infestation alone (Fig. 7a–d) and two of them were significantly induced by *S. frugiperda* attack alone (Fig. 7k–l). We found no significant effect of later *S. frugiperda* attack on *D. v. virgifera‐*induced volatile emissions (Fig. 7). However, the induction of the *S. frugiperda*‐induced volatiles was suppressed by early *D. v. virgifera* infestation (Fig. 7l). This result demonstrates that *D. v. virgifera* canalizes the root volatile production and renders roots unresponsive to leaf attack by *S. frugiperda*.

## Discussion

The sequence of arrival is increasingly recognized as an important determinant of plant‐mediated indirect interactions between herbivores (Viswanathan *et al*., 2005, 2007; Poelman *et al*., 2008; Erb *et al*., 2011a; Soler *et al*., 2012; Wang *et al*., 2014). However, the mechanisms leading to sequence specificity are not well understood. The goal of the present study was to identify the (mutually nonexclusive) behavioral and physiological mechanisms that may contribute to sequence‐specific effects. Our experiments show that leaf attack by *S. frugiperda* strongly reduces the attractiveness of roots for *D. v. virgifera* through changes in volatile cues. However, prior *D. v. virgifera* attack suppresses these changes and thereby maintains the attractiveness of the plants to *D. v. virgifera* larvae. This form of asymmetrical host acceptance behavior explains why *S. frugiperda* reduces the abundance of and damage by *D. virgifera* in the field only when arriving first on the plant (Erb *et al*., 2011a).

Several nonexclusive physiological mechanisms may explain why *D. v. virgifera* is repelled by *S. frugiperda*‐attacked plants only when arriving second. It is for instance possible that early‐arriving *D. v. virgifera* larvae change the behavior and induction pattern of *S. frugiperda*. However, we found no evidence for the presence of resistance feedback loops in our system: *S. frugiperda* damage remained unchanged by *D. v. virgifera* attack. Earlier studies demonstrated that *D. v. virgifera* root attack increases leaf resistance via ABA signaling under drought conditions; when plants were well watered, no negative effects of *D. v. virgifera* on *Spodoptera littoralis* growth were observed any more (Erb *et al*., 2011b). The maize seedlings in our experiments were supplied with sufficient soil moisture, which probably prevented potential feedback loops from occurring. Another explanation for the observed behavioral patterns is that *D. v. virgifera* may induce changes that strongly increase the attractiveness of the roots and override any negative changes that are later induced by *S. frugiperda*. By mixing volatiles from different plants, we tested this hypothesis on a behavioral level. Surprisingly, we found that *D. v. virgifera* rejected the volatile mix from a combination of plants that had been infested by *D. v. virgifera* and *S. frugiperda* separately. This is in stark contrast with the strong attractiveness of plants that were infested with *D. v. virgifera* and *S. frugiperda* sequentially and strongly suggests that *D. v. virgifera* does not produce an overriding attractive signal.

In contrast, our GC‐MS analyses provide clear evidence that *D. v. virgifera* canalizes the plant's root volatile response. Maize roots responded strongly to *D. v. virgifera* attack and produced higher amounts of several volatiles, including several products of the terpene synthase TPS23 which are strongly induced by *D. v. virgifera* (Köllner *et al*., 2008; Hiltpold *et al*., 2011) and attract the root feeder (Robert *et al*., 2012a). These responses were not altered by later *S. frugiperda* attack. By contrast, *S. frugiperda* attack induced a different set of compounds in the roots, including a yet unidentified nitrophenol, and this induction was fully suppressed by prior *D. v. virgifera* attack. These results demonstrate that early‐arriving *D. v. virgifera* canalizes the root metabolism in a way that makes it unresponsive to *S. frugiperda* attack. Canalization of plant responses by herbivores has been proposed to occur in a number of plant–herbivore interactions (Thaler *et al*., 2002; Viswanathan *et al*., 2005; Utsumi *et al*., 2010). For example, Viswanathan *et al*. (2007) found that tortoise beetle (*Plagiometriona clavata*) attack after flea beetle (*Psylliodes affinis*) attack of *Solanum dulcamara* did not alter the induced resistance elicited by the flea beetles. By contrast, tortoise beetle attack before flea beetle attack resulted in the disappearance of induced resistance. One possible explanation of canalization is negative cross‐talk between signaling pathways such that inducing one pathway may attenuate or repress other pathways (Koornneef & Pieterse, 2008; Erb *et al*., 2012). Furthermore, priority in occupying a plant resource may also result in physiological canalization, as resources invested into an initial induced response may not be available for investment into later induced responses (Stam *et al*., 2014). In combination with the behavioral experiments, these results suggest that the asymmetrical host acceptance behavior of *D. v. virgifera* is caused by physiological canalization.

In a previous study, we found that leaf attack by *S. littoralis* leads to a slight decrease in root ethylene production, and that adding ethylene back to the root system restores the attractiveness of the roots to *D. v. virgifera* (Robert *et al*., 2012a). Many herbivores increase local ethylene emissions of their host plants (Winz & Baldwin, 2001; von Dahl & Baldwin, 2007; Schäfer *et al*., 2011), and it is therefore possible that *D. v. virgifera* attack resulted in the reversal or canalization of the ethylene response of the roots. Unfortunately, ethylene emissions could not be measured in the current series of experiments. However, the presented findings suggest that *S. frugiperda* attack also triggers the release of repellent volatiles which are suppressed by *D. v. virgifera*. The escape experiment in particular shows that *D. v. virgifera* systematically moves away from leaf‐infested plants, and it seems unlikely that a reduction in ethylene concentrations alone can account for this result. Furthermore, the volatile mixing experiment suggests that the volatile blend of the roots of an *S. frugiperda*‐attacked plant overrides the attractive signal from a *D. v. virgifera*‐infested root system.

In our GC‐MS chromatograms, we found several volatiles that increased in the roots of *S. frugiperda*‐attacked plants. Elucidating their structure and bioactivity is an exciting prospect for future work. A recent paper identified methyl antranilate as a repellent for neonate *D. v. virgifera* larvae (Bernklau *et al*., 2016). Although methyl antranilate was not among the *S. frugiperda*‐induced root volatiles, it provides an interesting starting point to identify the volatiles that render *S. frugiperda*‐attacked plants repellent to *D. v. virgifera* larvae. One aspect that should be kept in mind is that root volatiles were measured by grinding root material and sampling the headspace of the ground samples by SPME. The advantages of this technique are its sensitivity and robustness. Its disadvantage is that it may result in the detection of volatile compounds that are not actually released into the rhizosphere by intact roots. Future experiments should therefore include *in vivo* sampling techniques to confirm the release of the newly detected volatiles into the rhizosphere (Ali *et al*., 2010; Hiltpold *et al*., 2011).

Host location and acceptance by herbivores are key processes in plant–herbivore interactions. Our results show that physiological canalization can have a strong, sequence‐specific impact on host acceptance by herbivores, which may result in strongly diverging herbivore damage and distribution patterns in the field. Our previous work shows that the repellent effect of leaf infestation on root herbivores is highly conserved across herbivore species and maize genotypes (Lu *et al*., 2016). Whether similar effects also occur in other plant species remains to be elucidated. Understanding the mechanisms that govern sequence specificity will allow for the integration of this phenomenon into current theory on plant‐mediated interactions and will facilitate future efforts to develop predictive ecophysiological models of multi‐herbivore dynamics on shared host plants.

## Author contributions

M.E., W.H. and C.A.M.R. planned and designed the research. W.H., L.H., Z.B. and C.A.M.R. carried out experiments. W.H., M.R.H. and M.E. analyzed data. M.E. and W.H. wrote the manuscript.

## Supporting information

Please note: Wiley Blackwell are not responsible for the content or functionality of any Supporting Information supplied by the authors. Any queries (other than missing material) should be directed to the *New Phytologist* Central Office.

**Fig. S1** Infestation by *D. v. virgifera* does not change aboveground damage by *S. frugiperda* larvae.
