# Supplementary material for: A mechanism for sequence specificity in plant‐mediated interactions between herbivores
Source: New Phytol. 2016 Nov 29;214(1):169–79. doi: 10.1111/nph.14328 (PMC6079637; doi:10.1111/nph.14328)

New Phytologist

***New Phytologist* Supporting Information**

Article title: **A mechanism for sequence specificity in plant-mediated interactions between herbivores**

Authors: Wei Huang, Christelle A. M. Robert, Maxime R. Hervé, Lingfei Hu, Zoe Bont and Matthias Erb

Article acceptance date: 11 October 2016

The following Supporting Information is available for this article:

Fig. S1 Infestation by *D. v. virgifera* does not change aboveground damage by *S. frugiperda* larvae. Relative and absolute leaf damage caused by *S. frugiperda* on plants with and without previous infestation by *D. v. virgifera* is shown. AG, aboveground *S. frugiperda* larvae infestation; BG>AG, belowground infestation followed by aboveground infesatation. Values are means ± 1 SE (n = 18).

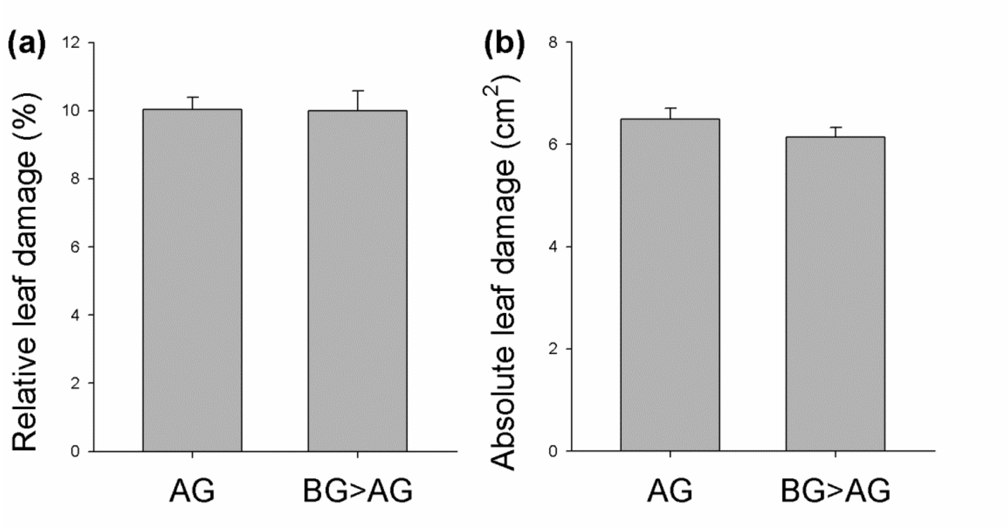

Supplement: Supplementary file 1 — Fig. S1 Infestation by D. v. virgifera does not change aboveground damage by S. frugiperda larvae. [file NPH-214-169-s001.pdf]
